# The experience of motivation and adherence to group-based exercise of Norwegians aged 80 and more: a qualitative study

**DOI:** 10.1186/s13690-019-0354-0

**Published:** 2019-06-07

**Authors:** Irene Vestøl Stødle, Jonas Debesay, Zada Pajalic, Inger Marie Lid, Astrid Bergland

**Affiliations:** 1 Oslo Metropolitan University, Faculty of Health Sciences, Pilestredet, PO Box 4, St. Olavs plass, 0130 Oslo, Norway; 2 VID Specialized University, Faculty of Health Studies, PO Box 184, Vinderen, 0319 Oslo, Norway

**Keywords:** Motivation, Adherence, Older people, Exercise, Physical activity, Salutogenesis, Comprehensibility, Manageability, Meaningfulness

## Abstract

**Background:**

Physical activity is crucial for public health; worldwide, across all age groups, exercise has been recognised as a factor that leads to improved health. However, many people do not engage in regular physical activity and hence miss the opportunity to achieve these significant physical and mental health benefits. With the benefits of exercise in mind, the aim of the present study is to describe the experiences of older people’s motivation for participating in and adhering to a group-based exercise intervention in a local community setting.

**Methods:**

A qualitative design was used in which semistructured interviews of three men and four women of an advanced age (81–92) were conducted; the participants described their experiences with their participation in and adherence to a long-term group exercise intervention programme in a community setting. Data were analysed using systematic text condensation and discussed in light of the salutogenetic theory.

**Results:**

Four main themes emerged from the data collection: (1) Experience of health challenges: A meaningful starting point; (2) Adherence motivated by increased life-manageability; (3) Comprehensibility through skilled instruction and (4) Social and professional support enhancing motivation. The participants; who had experienced negative changes to their health and function, as well as to their life situation, chose to sign up for the exercise groups and continued to participate throughout the entire intervention. Attending group exercise sessions meant positive changes to physical, mental and social functions enhancing the participants’ motivation to sustain their attendance and leading to positive behavioural changes that were important to their everyday lives.

**Conclusion:**

Essential factors for these participants’ decision to sign up for the exercise groups were the following: having important life areas connected to physical activity in their history and having belief in exercise as an effective way to restore function and coping; as well as having current experience of health challenges. Adherence to the group exercise was associated with better coping and the ability to fulfil roles and keep up with important life areas. Support from family, friends and professionals also contributed, both to the process of signing up, and adhering to this long-term intervention. The professionals’ skills and the way the instructor tailored group instruction were emphasized as very important to the participants’ adherence. Understanding of motivational factors for participation in and adherence to, exercise programmes is of great importance to older people, health professionals and society.

## Background

No matter where in the world, regular physical activity and exercise are crucial to the general public’s health and have been recognised as leading to good health and to keeping people across all age groups mobile and independent [1, 2]. Many people do not exercise regularly, and hence miss the positive effect exercise has on their health [1]. Worldwide, for those aged 75 and over, the proportion of people who do not meet the recommended guidelines is 75% [3]. This is turning out to be more of a problem because advances in medical care, healthier lifestyles and better access to family planning have contributed to increased average life expectancy age; thus, the number of older people in the world population has increased [4, 5]. When it comes to mortality, physical inactivity is the fourth highest risk factor worldwide [6] and physical activity and exercise play an essential role in maintaining independence, reducing the risk of falling, and allowing older people to live their lives well [7–9].

According to the World Health Organization (WHO), falling is the third leading cause of chronic disability worldwide, and approximately 30% of community dwellers aged 65 and older experience one or more falls every year [5]. Exercise programmes can reduce the risk of death and falls in older community-dwelling people [10]. In Norway, national authorities have focused on low-threshold exercise programmes through the Action-Plan for Physical Activity 2005–2009 – Together for Physical Activity [11]. This initiative has led to the organisation of group-based exercise programmes in many communities around the country.

Group based exercise has proven to be appropriate for increasing balance and muscle strength among older people [12]. In this context Rogers et al. [13] utilised a systematic review to understand why older people joined and continued to attend Tai Chi exercise groups. Based on 37 studies, the main factors influencing recruitment and participation were found to be social support in terms of being part of a group and maintaining and improving the health of participants, along with improving their overall quality of life. Other studies focused on positive emotions, the experience of support from fellow participants [14–16] and the influence of a good instructor, for example, in tailoring the exercises to the individual participant’s needs [14, 15]. Also, the belief that exercise can improve both one’s physical and mental health [8, 14–16] appears to be a motivator for starting to exercise. A high degree of self-efficacy, which is the belief in one’s capabilities to organise and execute the course of action required to produce certain attainments [17], has also been reported to motivate the recruitment and continued participation in fall prevention exercise [8, 15, 16].

## The conceptual framework

In Antonovsky’s salutogenetic framework, a central term is sense of coherence (SOC), which is connected to a person’s fundamental history and important life areas. The definition of SOC is the extent to which one has a pervasive and enduring, though dynamic, feeling of confidence that life’s events are comprehensible, manageable, and meaningful [18–20]; this term might help us understand how to motivate elderly persons to attend exercise programmes. In addition to SOC, another central term in the theoretical model of salutogenesis is general resistance resources (GRR). The concept GRR represents biological, material and psychosocial factors that make it easier for people to perceive their lives as consistent, structured and understandable and might be of importance regarding motivation for exercise participation. Typical GRR are money, knowledge, experience, self-esteem, healthy behaviour, commitment, social support, cultural capital, intelligence, traditions and views of life [21].

The value of salutogenesis has been demonstrated in health behavior studies [22, 23], and, according to Løvheim et al. [24], sense of coherence (SOC) represents a theoretical construct that has been used to explore factors that influence coping with life events in old age and the stronger SOC, the better perceived health. Understanding individual characteristics that motivate older people to surmount barriers and adopt behaviors can inform us in choosing approaches to better and increased health behaviors in old age. Furthermore, health workers, practicing from a SOC orientation, can strengthen individuals’ GRR to promote and reinforce a SOC [25]. According to Langius et al. [26], motivation is positively related to SOC [26].

Antonowsky’s theory of salutogenesis is used as a framing of the discussion of the findings in the current paper.

*Motivation* can be defined as the forces that initiate, direct and sustain behaviour [27]. This motivational perspective has emerged from empirical research conducted on young people. Vallerand and O’Connor [27] distinguished motivation as either intrinsic, self-determined extrinsic, nonselfdetermined extrinsic or amotivation; however, we will use only the concepts of intrinsic and self-determined, extrinsic motivation in the current study. Intrinsically motivated behaviours are engaged in for their own sake – for the pleasure and satisfaction derived from their performance [28]. Self-determined, extrinsically motivated behaviours are valued by the individual and are perceived as being chosen by oneself. The behaviour is extrinsic because the activity is not performed for itself, but rather as a means to an end [27].

*Adherence* can be understood as the extent to which the individual’s behaviour corresponds with caregivers’ recommendations and follows the mutual agreement to achieve established goals [29]. The concept of adherence includes autonomy for the person receiving the recommendation, allowing him or her the independence to decide to follow the recommendation or the mutual agreement. Because adherence to an exercise programme often involves a behavioural change, the addition of support for this process might provide a valuable contribution to adherence. Adherence to exercise programmes is fundamental to achieving positive outcomes from the exercise.

To the best of our knowledge, no previous qualitative study has explored participation by community-dwelling older people over the age of 80 [30]. Knowledge about these motivational factors could be helpful for health workers in stimulating participation and adherence to exercise interventions among elderly persons.

Therefore, the ambition of the current paper is to highlight what motivates older people to participate and adhere to group exercise programmes in local community settings.

## Methods

### Intervention, recruitment and participants

The study has a qualitative, descriptive design that is based on a phenomenological approach [31, 32]. Understanding the participants’ perspectives based on the descriptions of their motivational factors requires the ability to openly explore the participant’s lived experiences.

The general intervention programme had a nine months’ duration from September to May, with two weekly exercise sessions led by instructed and skilled physiotherapists and occupational therapists. Ten different groups used the same exercise programme in different parts of the community at the time. The intervention programme was built on elements from The Otago Exercise Programme (OEP), which is a strength and balance retraining programme designed to prevent falls in older people living in a community setting [10]. Four times every six months, the exercises were replaced by lessons in physical activity, motivation, nutrition and restitution.

The inclusion criteria for participating in the intervention were that the participants were home dwelling, aged 65 or older, able to understand the instructions, motivated, able to walk with or without a stick or walker and able to transport themselves to the training locality.

For the inclusion in the current study, the leader of the intervention helped find informants by asking all the group leaders to ask for participants in their respective groups. Originally, we asked for both informants who had low and high grades of adherence, but none of the participants with low adherence wanted to be interviewed. Among the participants with high levels of adherence, we recruited a purposeful sample [33] of participants 80 years of age or older. We asked for informants who varied in age, gender and group-affiliation to provide richness and variation in the data covering differences among the participants and the differences connected to instruction and locality. The fact that the intervention was conducted for a long time, ensured that the participants had lots of exercise-sessions to tell stories about. The recruitment-process was difficult, but in the end, we had seven participants who wanted to share their stories. After finishing five interviews, no new concepts or themes were emerging. After two more interviews, we realised that these two participants did not bring in new substantial information. The seven included informants contributed rich descriptions from various sides of the phenomenon of motivation for and adherence to group exercise.

### Semistructured interviews/data collection

Using individual semistructured interviews that were conducted in the participants’ own homes, where just the interviewer and the participant were present, the data were collected. The interviews lasted between 30 and 90 min. A semistructured interview guide [34] that contained main questions was used; this was supported by follow-up questions that helped to initiate reflections and obtain descriptions of the participants’ experiences of participating and performing exercise.

Examples of the questions asked were as follows: Would you please tell me about what made you sign up for the exercise group? Would you please tell me what made you continue to attend the groups for such a long time? Would you please describe the events from the group exercise that encouraged you to continue exercising?

All the interviews were audio recorded and transcribed verbatim by the first author.

### Data analysis

The data were analysed using a systematic text condensation process [35]. The analysis was inductive and grounded in the actual data, and categories were generated from the interview text throughout the analysis. By comparing the individual participants’ descriptions, we searched for patterns of commonalities and differences in the participants’ experiences. The analysis followed Giorgi’s phenomenological method [31], as modified by Malterud [36]. This form of analysis is theoretically anchored in the phenomenology and is based on the interviewees’ lifeworld as they experience them [36]. The analysis started with the first author engaging in a naïve reading of all the transcripts several times. Next was to the process of capturing the key concepts and thoughts; to do this the text was read in depth, word by word, to extract meaning units, focusing on the experience of performing exercise and the motivational factors that elderly persons pointed out as being the most important to their participation and adherence to the nine-months’ long group exercise programme. After this step, all the extracted meaning units were coded. The coded meaning units were grouped into subcategories and categories using a coding scheme. Finally, the analysis resulted in descriptions that fell into four categories. During the analysis, the meaning units, codes and categories were discussed among two of the authors; the analysis was mainly performed by the first author and validated by the co-authors, who followed every step in the analysis and read and commented on the analysis. The steps in the analysis process are shown in Table 1. To ensure the rigour of the analysis, all the authors read the final version of the analysis and the categories. Also, quotations are used to illustrate the findings to show the validity of our interpretations.

**Table 1:** Overview of the steps in the analyzing process

Meaning unit	Condensed unit	Code/theme	Subcategory	Category/heading in result chapter
.. and so the fact that I had my legs. I was scared to death that I should end up in a wheelchair, really. Yes, that was very close to happening because they were almost ruined by arthrosis, so I went to the gym with these terrible legs, I had to stop the long walks, I couldn’t do that anymore.	I exercise in the group instead of walking long distances, to prevent my legs from getting worse	Bad knees the motivation to exercise	Disease is the reason for starting the exercise-group	Experience of health challenges: a meaningful starting point
So, doing the dishes...When I have to stand for a while when I’m doing the dishes after dinner for example, with pots and stuff, I had to rest…and that went away. Suddenly I realised that… good heavens! Now I’m done and I didn’t need a break!	I no longer need to take a break when I’m doing the dishes!	Satisfaction through better coping	Feeling of control/coping	Adherence motivated by increased life-manageability
Well, I believe it is mostly because of the instructors. They know all about this and they can guide each one of us. Me, I have things I do wrong, so the instructor helps me do it right. But it’s also having someone who can start the exercises and can give advice.	It is important to be supervised by persons with knowledge	The instructor is important for the participants’ motivation	The instructor plays an important role	Comprehensibility through skilled instruction
(…) And I have a son who is a doctor and a daughter-in- law who is a specialist in geriatrics, in fact, so they give me advice. Especially my daughter-in-law tells me to exercise and that it is not dangerous even if it hurts	Competent people in my family encourage me to exercise	Support from family is important		Social and professional support enhances motivation

Lincoln and Guba discussed the following criteria for trustworthiness; credibility, confirmability and transferability [37]. To increase trustworthiness during the analysis, a coding scheme was used [38, 39]. In striving for trustworthiness and credibility, reflexivity was used in the analysis, and the authors strove to become aware of their preunderstandings and their influence on the emerging findings, as well as in how well the categories represented the data. The categories were discussed thoroughly to detect the differences between-, and similarities within the categories. In striving for credibility, the selection of participants, the data collection and the data analysis are presented as thoroughly as possible in the present study. Here, confirmability is the degree of neutrality or the extent to which the findings are shaped by the respondents and not the researchers’ bias, motivation or interest. To ensure concordance between the content of the interviews and our themes, we have illustrated the subthemes using quotations that are labelled with a code referring to the person who made the statement. All the authors made explicit their preunderstanding and existing knowledge about the context. The first author, a female, registered physiotherapist who performed the interviews and transcriptions, was also aware of the physiotherapist lens that she possesses.

### Ethical perspectives

The Data Protection Official at the Norwegian Centre for Research Data approved the study in a letter dated 08.12.2011, reference number 28652. The participants were given written and verbal information about the objective of the study prior to the interviews.

Written consent to be interviewed, recorded and being part of the whole study, was also obtained. The participants were informed that the information given would be confidential and that they were free to end their participation at any time and without any negative consequences for doing so.

## Results

Seven people agreed to participate in the current study (Table 2). The participants (age 81–92 years) had all been participating in the first season (sept-may) of the intervention, and six out of the seven were still exercising in a group when the interviews took place. Both genders were represented (see Table 2) and the informants were recruited from six out of 10 different exercise groups in the local community by key persons leading the intervention. According to these key persons of the intervention, all informants had attended the group exercises with a high grade of adherence. This was confirmed in the interviews when the participants mentioned that they only missed an exercise session in the case of acute illness or very important appointments such as hospital check-ups. At the time of the interviews, only one participant lived with a spouse. Two had their spouses in a nursing home and the remaining four were living alone after losing their respective spouses.

**Table 2:** Demographic information of participants

Informant	Gender	Living alone	Health challenges
1	F	Yes	Dizziness, pain after hip prosthesis operation with complication fracture after operation in leg
2	M	With wife	Pulmonary embolism with complications
3	F	Yes	Back pain, heart disease, multiple fractures
4	F	Yes	Knee replacement both sides
5	M	Yes	Psychiatric trauma/depression/arthrosis hip
6	M	Yes	Polynevropathia
7	F	Yes	Depression, leg pain

Four overreaching and interrelated themes emerged from the interviews regarding the participants’ experiences with participation in the exercise groups: (1) Experience of health challenges: A meaningful starting point; (2) Adherence motivated by increased life-manageability; (3) Comprehensibility through skilled instruction; and (4) Social and professional support enhancing motivation.

## Experience of health challenges: a meaningful starting point

All the participants reported that they had experienced major changes to their health situation prior to the intervention, both physically and psychologically. Some also had experiences of dear ones’ diseases and death, and this also had an impact on their overall life situations. It seems as though these changes contributed to the decision to sign up for the exercise groups.

The health challenges the interviewees experienced had worsened their ability to walk. All the participants had positive experiences with physical activity earlier in their working lives or with different forms of walks in their spare time. They reported that some of the challenges and demands posed by their current lives made investing energy in group training worthwhile. The participants seemed to embrace their life challenges with commitment and engagement rather than perceiving them as burdens that one would rather do without.

The thought about joining some form of exercise programme seemed to have matured in the informants prior to the time they received information about the exercise-groups, so the information came at a time they were prepared to begin a training schedule. A female informant described this in the following way when she first learned about the groups:

*I became very happy when I first saw the advertisement in the newspaper. So, I thought that this is something I want to sign up for. Yes. And it didn’t last long before I heard from my friend in the neighbourhood, that she also wanted to come along. So, we signed up at the same time and it was very fun. (female, 81)*

The changes in the health situations that the participants experienced also led to changes in their living conditions, at least for some, such as for the following man who had just moved into an assisted-living accommodation:

*So, this is my situation, and when I came in here in July last year, they announced that they were going to start this event. I signed up right away. I was actually the first person to sign up, I think. I saw it advertised in the newspaper, and it appealed to me right away. It was strange how few people signed up, only three or four persons who were living in the nursing home. (male, 86)*

Several participants stated loneliness and depression as the reasons that motivated them to join the groups and said that the social element played an important role for them. The fact that the exercise was organised in groups and had the potential to fulfil these social needs, seems to have been an essential factor to motivate the participants to sign up.

The feeling of loneliness after the loss of a spouse or other loved ones was felt by several participants. A 92-year old woman felt lonely after her spouse and several others in her network had passed away; she said the following:

*Yes, it was well-known that I had a period when I was not all that good. My husband died three years ago, so I was very lonely, and I was sad and sat at home and didn’t want to go out ... however, I have not been very social all my life, I should say. Moreover, my husband had few friends left here, so I felt somehow so alone. (…). And then the kids said; ‘you need to get out among people’, which I think was good advice (female, 92*)

## Adherence motivated by increased life manageability

All the participants showed a high degree of motivation and adherence throughout the intervention programme. They indicated that the primary motivating factor for their high level of participation and adherence was that they had expected- and did indeed experience- that the exercise increased their life manageability. Their beliefs about difficulties or challenges as being solvable and their experiences that the exercise increased the resources at their disposal so that they could become more able to meet the demands regarding social participation, mental health and physical performance both strengthened their motivation and adherence to the programme. The exercise contributed to their experiences of coping with tasks that were important to the participants. One participant who suffered from a chronic progressive disease did not experience improvement in his physical condition but was nevertheless the one who exercised the most because of his belief that exercise could slow the progress of his disease. The other participants reported that their arms and/or legs had become stronger. A woman noticed that it was easier to get out of bed in the morning. Other participants said their balance had improved and that they fell less frequently. Some also mentioned that they could walk a longer distance than before the training started, which meant a lot to them because several participants reported that they especially enjoyed walking. Two of the men had to walk some distance to get to the exercise locality. They managed well through the winter, and one of them described the change in his walking distance as follows:

*I had a brother in a nursing home nearby, but to walk all the way from here and up there, even if I used the walker, I could not have done that before. [...] I can obviously do it now, and even though the way home may be long, I can still do it. [...] and because of that my muscles need to become even stronger, so I’m going to continue in these groups till I get it right. (male, 83)*

Many participants described specific tasks at home or critical areas in their lives where they had noticed a distinct change in manageability after they had started the exercise groups. A widow had a cat that she always had to let out or in, and this kept her moving about in her apartment. It became easier when her balance and the strength in her legs had improved. One participant said that now, it was possible to do all the dishes after dinner without taking a break. Another woman who lived on her own described how her arms had become stronger after she had started exercising:

*However, I told them down there, and they laughed themselves sick. It started when I said that the litre of milk had become so much lighter! What did you say, a litre of milk? ‘Do you think they fool you on the amount?’ they said. Yes, I had to wonder. When I took the milk carton out of the refrigerator earlier, I had to use both hands. -I felt I couldn’t really keep it steady. Suddenly, it didn’t matter at all. I didn’t even have to think about it. [laughter]. So, exercising has helped me a lot. (female, 91)*

The oldest woman described how meaningful it was for her to be able to do her housework, which had always been her main task in the family:

*Yes, that’s while I have been doing this [exercising], taking control, and I really think that I can manage on my own now, and today, while the instructor talked about the fact that you shouldn’t overthink about your exercise every day because there’s exercise in everything you do – the chores – and so you use your body, you use your whole body. Moreover, I can lie on the floor and crawl, I can kneel and wash the floor, I can hang up curtains and clean the windows, and all this I do not want to lose because it is such a good feeling to be independent, to disentangle myself and to not have just to sit there and wait for someone to help me. (female, 92)*

Managing practical, physical tasks triggered good feelings for the participants. They felt that they were in better mental shape as a result of their participation in the exercise groups. Just meeting others and being in a social context were seen as tangible improvements to their mental health. This indicates there was a motivating factor of improved life manageability.

## Comprehensibility through skilled instruction

The instructors/ therapists were described as *supportive* and *inspiring*, and the participants said that these instructors/therapists -*see their patients*. Furthermore, the ability to create clarity and structure, as well as to present the rationale for the programme, was necessary for the informants’ participation and adherence to the programme. The participants reported that the instructors contributed to the comprehensibility of the exercise and improved the participants’ understanding and insight of the programme. The instructors had great knowledge about the exercise and muscles because they were all trained physiotherapists or occupational therapists. When the participants had different performance levels and diseases, the instructors’ specific knowledge of this training was particularly important for participants’ confidence in the instructors’ advice and guidance. If anyone asked for additional help, they were given the adaptation they needed. This ability of tailoring the exercise to the participant was expressed regarding the participants’ anxiety about over-exerting their hearts during the exercises, as in the case of this 91-year-old woman:

*We had two excellent instructors (…) We were told to do our best and then not. If we got exhausted, we were told to take a break. Moreover, that was alright not to be pushed too hard. For example, when we threw balloons between each other. It was very exhausting for me. So I was a bit careful with that exercise. (female, 91)*

Tailoring was also expressed regarding the participants’ difficulties with particular unique movements such as in the following case:

*There are some participants in the group who have trouble with their knees, and they cannot do all the exercises that I can, so they try to find other ways they can do it. However, it is the instructor who takes responsibility to help them. They are very clever to guide if some of us don’t manage to do it right. (female 81)*

The instructors’ good relational skills, the fact that they give important individual feedback, are nice people and have relevant knowledge all contributed greatly to maintaining the motivation to exercise for the participants. One woman received the following encouragement from her instructor:

*Yes, it’s perhaps the instructor as well, for it was something we talked about. She said, ‘I saw you one day on the way to the store with the walker, do you go to the store and shop?’ Yes, I do, but what was it she said ... yes, I still take the bus back*. *-And then .. What was it she said … I cannot remember it quite ... you will be able to walk both to the shop and home again when you turn 100! (female, 92)*

A male participant put it the following way:

*I compete with myself, and if I get a word of praise from one instructor, I think that’s nice; it’s the same with the other. However, I think the reason is quite essential. And the fact that the instructors are, first, very astute and also very lovely, even charismatic, [...] can help to make things a little more joyful while we are doing the exercises. (male, 83)*

## Social and professional support enhancing motivation

Most of the participants were in regular contact with their doctors/GPs and quoted the doctors’ support for their participation in the group approach as being important to them. Some mentioned that their doctors had curbed their activity, considering it to be too much, or that their doctors had said that they should not overexert themselves because of heart disorders with heart disorders. One doctor had been struggling to motivate his patient to see a physiotherapist, but when she started with the exercise group he was pleased with that:

*My doctor nagged me to make sure I was going to do some physical therapy because of my problems, but I didn’t feel like it, so I thanked him but refused. However, he fussed about it several times, so I thought that I could do this instead -it’s got to help a little -so I started with this [the exercise groups]. (…).*

*Moreover, it was just ‘spot on’, he said (the doctor), ‘and it’s just tailored to fit you, so you don’t over-exert yourself, for you should not [do that]’ … And now he boasts about it, because I used to have my blood pressure taken two or three times a day, but since I started to exercise, he hasn’t mentioned that at all. (female, 91)*

One informant had a relative who was a doctor, and she had said that he needed to exercise even if it was strenuous. Indeed, close relatives’ support and encouragement are important for motivation and adherence. One woman said that her daughter never records the times of her visit during the exercise, because she knows how important it is to her mother.

*They – friends and acquaintances, my daughter and everybody – know they must not disturb me during the hour when I go to my exercise class, therefore! [...] but those with whom I do it smile a little when I say that this is something I really like to do and the fact that we do this together (in the group) is great! Exercising at home on your own is not so easy. (female, 81)*

Praise, encouragement- and even applause from fellow participants in the group situation were also recognised by many individuals as essential for participation. The fact that the exercises were done in groups where the participants could meet others in a similar situation was essential to staying motivated.

One specific exercise was highlighted as unique for its encouragement: namely lying down and getting up from the floor. Several participants described how they had improved here and that the encouragement from fellow participants was especially important. One man (83) put it in the following way:

*Things that have been a little difficult for some, for example, what I had already learnt at the hospital, to lie down on a mat and then get up again from the mat, and you are advised to place a chair next to you if you need to use it, but if you get up off the floor without using the chair, then you do not exactly get ovations, but something close, and you feel a little proud even if you are an old man! (male, 83)*

Several participants gave examples of this effect when citing other exercises, talking about when they had received positive comments from the other participants in the group on the way they managed the exercises. The oldest participant, a 92-year-old, initially had doubts as to whether she was too old to be in the group, and now, she says how the praise from the younger participants has been positive for her:

*Yes, I don’t know how old they are; they have not said how old they are, but they are younger than me, and they say, ‘You sure are good!’ And I think that’s quite fun, yes, a bit of fun. (female, 92)*

This appeared to be important to the person who provided the encouragement as well because it seemed to make an impression on them -here seeing others manage the exercises- and this also encouraged the others in the group who witnessed it:

*…and when we are down on the floor and are told to get up and lie down, you know, the person who needed a little help from the coach. She manages to get down on the floor! -It’s amazing because she also manages to get up again and it impresses the rest of the gang and we applaud! (female, 81)*

## Discussion

The individuals in the current study reported negative changes in health and function, as well as their life situations, as the major driver to their high level of participation and adherence to the exercise groups. Participants reported that attending group exercise sessions meant positive changes in physical, mental and social functioning, enhancing their motivation to sustain their attendance and leading to positive behavioural changes that were important in their everyday lives.

Our findings fit the salutogenetic perspective, which indicates the importance of the individuals’ resources used for understanding, managing and making sense of the situation with which they are faced [21, 40].

This is in line with our results that align with different reviews and meta-syntheses, all of which have concluded that exercise for community-living older people increases functional capacity, wellbeing and health-related quality of life [41–45]. WHO considers that *active ageing* is a key concept that allows people to realize their own potential and to live through their own ageing as a positive experience with continuing opportunities for health, participation and security [46], and this corresponds with our results. The empowering feedback the participants received from other members of the group, the instructors and other influential persons in their lives contributed to their continued participation and motivation. These findings are in line with those of Duncan et al. [47], who stated that engaging in regular physical activity is an important part of a healthy lifestyle. In line with the results of other researchers, our participants reported that exercise increased their strength, balance and endurance [10, 48].

## Personal history leads to motivation for exercise

For many older people, the hardest thing is taking the initiative and getting started with the life-style changes that regular exercise can represent [15]; this was expressed by participants in the current study, and they also expressed their need for support from other significant persons, such as their doctors and family members. Antonovsky [18, 20] pointed to the importance of the person’s history in using a salutogenetic perspective, stating that a deeper understanding of people’s complex lives is a prerequisite for understanding why some people are able to change behaviour in order to move towards obtaining a better health state. The participants’ pleasure in being able to use their bodies, to go for walks or perform housework seem to have been an important factor that contributed to the internal forces that helped them choose to sign up for the exercise groups. In accordance with our motivational framework, this is behaviour that may be explained as self-determined extrinsically motivated because the activity is chosen by the person and is performed as a means to an end, namely to restore a function that allows physical activity of a certain kind [27].

In Antonovsky’s salutogenesis theory, a person’s behaviour is related to the strength of SOC, which is connected to the person’s fundamental history and important life areas. All informants in the current study talked about being active as an important part of life for them throughout the whole life cycle. They talked about an active childhood, doing housework and going for walks, both short and long. Because of their age (81–92), they all spent their childhood and youth using their bodies for different tasks that were common parts of their everyday lives, and they seemed to have learnt to appreciate that. They all had been in a situation where they thought it was necessary to make some adjustments. From this perspective, SOC acts as a classic moderator for life stress [18, 20].

In general, the ability to cope with stress and change depends on the extent to which the world is perceived as comprehensible, manageable and meaningful [18, 20]. In the current study, the stressors the informants experienced were health challenges and the loss of their bodily capabilities, as well as the loss of loved ones. A person’s health challenges and overall life changes become a part of his or her history and life situation, establishing the base for the person’s SOC [18, 20]; this seems to have led to the participants’ decisions to attend the exercise groups. Negative changes in the participants’ lives led to adjustments that they believed would give them positive outcomes.

## Adherence by positive changes – through strong SOC

WHO has defined adherence as *the extent to which the patient follows medical instructions* [49]. In the present study, adherence is the extent to which the person continues to attend the group exercise programme twice a week throughout the intervention period and does so while displaying a high level of participation. The participants we interviewed found the motivation to sign up for the exercise groups, and from a salutogenetic perspective, we suggest that a strong SOC may be a contributing factor for this. The reasons given for staying in the programme when the participants had experienced the exercise programme, seem to have confirmed and helped maintain this strong SOC. Through group exercise, the current study’s participants experienced subjective improvements in their own physical capacity, coping and, quality of life, which confirmed their initial beliefs. Furthermore, they described their gains from the exercise as their arms and legs becoming stronger, feeling more flexible, it is becoming easier to get out of bed in the morning, their balance improving and being able walk longer distances. The ability to hang up curtains and clean the windows, meet others and be in a social context were all tangible improvements for the participants. Given the participants’ histories in which activities connected to physical activity were important hence creating a strong SOC the experience of taking part in the exercise groups and the bodily recognition of improvements likely supported the strong SOC they possessed.

The salutogenetic component of *meaningfulness* measures the extent to which people feel that life is understandable and that some of life’s problems are worth resolving [18, 20]. Through their experience of major improvements, the participants found that participating in the exercise groups was worth their effort, and this gave them further motivation to continue. The importance of functional improvements as a further source of motivation is also supported by earlier qualitative research. De Groot and Fagerstroem [16] found that functional independence and the maintenance of one’s walking ability were important motivating factors for participation and adherence. This behaviour, which is based on the experience of functional improvements, is extrinsic motivation because the activity is chosen by the person and is performed as a means to an end [27]. The participants in the current study also talked about exercise triggering good feelings on a psychological level, which in and of itself was a positive accomplishment. This might indicate that the participants experienced self-determined extrinsic motivation, which is intrinsically regulated but is performed to reach a goal outside of the activity [27].

The link between coping and health is crucial in Antonovsky’s [18, 20] framework. Physical activity and adhering to health-related recommendations are mentioned as coping strategies that can promote health; in addition, there is a strong probability that people with a strong SOC are more likely to choose health-promoting behaviour when first exposed to stress. Furthermore, the awareness of the physical benefits gained from regular physical activity are important factors in overcoming the barriers to engaging in physical activity [3]. This aligns with a study by Cederbom et al. [50] in which older individuals expressed the importance of activities in everyday life and the role of health care professionals in supporting and promoting their activities [50]. Antonovsky [18, 20] wrote about the feelings of hope and excitement that can create motivational and cognitive foundations for action when handling an instrumental problem. Vallerand and O’Connor [27] note that an individual’s feelings of competence and /or self-determination are intrinsically rewarding and that the activities that give these kinds of emotions are likely to be performed again [27]. Intrinsically motivated behaviour is characterised by pleasure and satisfaction in the activity itself and is derived from the need for self-determination and the feeling of competence [27]. The sample in the current study talked about how participation in the exercise groups triggered feelings of joy and happiness, along with the pleasure of being part of a group. This seems to have given a high level of intrinsic motivation to participate and adhere to the exercise programme. Here, to promote exercise among older people, enjoyable and familiar activities are preferable [51]. The beliefs the participants had regarding positive changes when joining the exercise groups were confirmed through participation, and the gains they achieved seemed very important to their overall functioning on numerous levels.

## The instructor facilitates motivation and adherence

The authors of a systematic review that focused on the factors associated with better adherence rates concluded that adherence rates are generally higher in supervised programmes [52]. The participants were encouraged by the instructor’s support and the tailoring of his/her instructions to their needs and by the whole group’s care and applause for their efforts. Antonovsky [18, 20] described how health personnel could to increase a person’s SOC levels by creating internal coherence, balancing stress and encouraging self-determination. It seems as though the current study’s instructors’ methods for leading the groups and taking the individual’s needs into account support and even increase the participants’ already strong SOC, and that this leads to maintained motivation and adherence. Supported by other studies [14, 15, 53], our findings suggest that the instructors play an essential role in facilitating motivation and adherence by acting as expert consultants and supporting participants while they orient themselves and navigate through the group sessions and their everyday lives. The instructors played a central role in helping the participants reflect upon and interpret their bodily experiences and in building up their confidence, thus making the new exercise situation more comprehensible, meaningful and manageable. The findings of the current study indicate that the instructors provided the participants with the cognitive, emotional, physical or material resources that could help them cope with the challenges in their daily lives [21, 54]. Navarro et al. [55] found that older people are more likely to accept exercise programmes if they do not need to exercise alone and if the programme has a good structure, provides a positive experience and, is performed in an environment suitable to the individual, which supports the findings of the current study.

Our informants stressed the encouragement that they and their peer participants had been given when it came to the decision-making, goals, opportunities and, coping resources that occurred in the group session. The literature has indicated a salutogenetic orientation in professionals’ dialogues with patients can contribute to enhancing the patients’ quality of life and decrease their psychological distress [54]. In line with our results from another qualitative study on exercise, older individuals mentioned that the supervisor must be a competent, confident and tolerant facilitator when directing an exercise group [56].

This fits in with other studies that have recognised the importance of a physician’s recommendation to older adults to engage in physical activity [55].

The fact that the groups in the current study were instructed by trained occupational therapists or physiotherapists seems to have been of great importance to the participants’ trust in the guidance and advice given by the instructors. Information given in a group setting can lead to better understanding and SOC.

## Strengths and limitations

In qualitative research, the goal is to enhance the understanding of the phenomenon being studied [57]. This current study gives voice to older people’s motivation and adherence to participate in group-based exercise. This is important knowledge when tailoring group-based exercise. The findings of the present study are, however, limited by the specific demographics of the small sample size and their geographical location; hence, the findings cannot be generalised, but may be transferred to similar situations or people [57]. Only seven people aged 81–92 years were interviewed in the current study and they all reported a high level of participation adherence. We failed to recruit participants with a lower level of adherence, so the information from this group is hence not reflected in the current paper. Because of the small sample, the current study cannot cover all the experiences that build into the motivation for participation and adherence to exercise groups. However, we still believe that these individuals’ insights and descriptions of their own motivations, can contribute to better understanding of the phenomenon being studied.

The researchers’ preconceptions may have influenced the research process [33]. The authors of the present paper have backgrounds within physiotherapy (IVS and AB), nursing (JD and ZP) and public health and ethics (IML). Throughout the research process, we were conscious that our preunderstandings and backgrounds might have had an impact on our data interpretations. We tried to interpret the data as open-mindedly as possible and have strived to provide a transparent description of the path from the data to results. The involvement of multiple researchers with different backgrounds may also strengthen the design of a study because they can supply and contest each other’s statements. In addition, the interviewer did her best to put the participants at ease and to listen emphatically and carefully to their stories.

## Conclusion

Having important life areas connected to physical activity in their histories and holding beliefs in exercise as an effective way to restore function and coping seemed to have made the participants choose to sign up for the intervention groups. From a salutogenetic perspective, the participants had, a strong SOC, which is indicated by the individuals’ resources for comprehending, managing and making sense of the situation with which they were facing. The strong SOC was confirmed through reported experiences of physical, psychological and social improvements in the form of better coping and the ability to fulfil roles and keep up with important life areas. Skilled professional and individually tailored instruction was emphasised as important for the participants’ adherence to the group exercise.

Understanding the motivational factors for participation in and adherence to exercise programs are of great importance to older persons, health professionals and society when tailoring interventions.

## Data Availability

Data generated and analysed during this study are included in this published article. The raw-data are in the Norwegian language and are available from the first author on request.

## Abbreviations

GRR: General resistance resources
OEP: Otago exercise programme
SOC: Sense of coherence
WHO: World Health Organization
